# Postoperative serum magnesium levels as a predictor for the need for calcium replacement after total thyroidectomy: a prospective study

**DOI:** 10.20945/2359-3997000000581

**Published:** 2023-01-17

**Authors:** Carlos Segundo Paiva Soares, Cristiano Claudino de Oliveira, Katia Hiromoto Koga, Sonia Marta Moriguchi, Simone Antunes Terra, José Vicente Tagliarini, Gláucia Maria Ferreira da Silva Mazeto

**Affiliations:** 1 Universidade Estadual Paulista Faculdade de Medicina de Botucatu Departamento de Oftalmologia, Otorrinolaringologia e Cirurgia de Cabeça e Pescoço Botucatu SP Brasil Departamento de Oftalmologia, Otorrinolaringologia e Cirurgia de Cabeça e Pescoço, Faculdade de Medicina de Botucatu, Universidade Estadual Paulista (Unesp), Botucatu, SP, Brasil; 2 A.C. Camargo Cancer Center Departamento de Anatomia Patológica São Paulo SP Brasil Departamento de Anatomia Patológica, A.C.Camargo Cancer Center, São Paulo, SP, Brasil; 3 Universidade Estadual Paulista Faculdade de Medicina de Botucatu Departamento de Medicina Nuclear Botucatu SP Brasil Departamento de Medicina Nuclear, Faculdade de Medicina de Botucatu, Universidade Estadual Paulista (Unesp), Botucatu, SP, Brasil; 4 Universidade Estadual Paulista Faculdade de Medicina de Botucatu Departamento de Patologia Botucatu SP Brasil Departamento de Patologia, Faculdade de Medicina de Botucatu, Universidade Estadual Paulista (Unesp), Botucatu, SP, Brasil; 5 Universidade Estadual Paulista Faculdade de Medicina de Botucatu Departamento de Clínica Médica Botucatu SP Brasil Departamento de Clínica Médica, Faculdade de Medicina de Botucatu, Universidade Estadual Paulista (Unesp), Botucatu, SP, Brasil

**Keywords:** Calcium, hypocalcemia, hypoparathyroidism, magnesium, thyroidectomy

## Abstract

**Objective:**

Our aim was to assess the ability of serum magnesium (Mg), measured on the first postoperative day (Mg1PO), to predict the need for calcium (Ca) replacement in patients undergoing total thyroidectomy (TT).

**Subjects and methods:**

Eighty patients undergoing TT, with Mg1PO and PTH dosage in the first (PTH1h) and eighth (PTH8h) hours after TT, were evaluated for the need for Ca replacement. Data were evaluated by uni/multivariate logistic regression and Receiver Operating Characteristic (ROC) curve.

**Results:**

32 patients (40%) required Ca replacement. Median PTH1h, PTH8h and Mg1PO were higher in the no replacement group: 17 versus (vs) 3 pg/mL (p < 0.001), 18.2 vs 3.0 pg/mL (p < 0.001) and 2 vs 1.6 mg/dL (p < 0.001), respectively. Mg1PO was the isolated predictor for this replacement (odds ratio = 0.0004, 95% confidence interval: 0.000003-0.04; p = 0.001), with the cut-off value of 1.8 mg/dL showing sensitivity and specificity of 78.1% and 87.5%, respectively.

**Conclusions:**

In this group of patients, serum Mg1PO was the isolated predictor for the need for Ca replacement.

## INTRODUCTION

Hypocalcemia is the commonest postoperative (PO) complication seen in total thyroidectomy (TT). Its prevalence substantially varies between different services, partly because there is no, mainly laboratorial, consensus on its exact definition (1). Post surgical hypocalcemia leads to higher risks for the patient, hospital stay, and costs (2). Consequently, various studies have been conducted looking for early and safe predictors for patients at risk of developing this complication.

The main cause of PO hypocalcemia in TT is hypoparathyroidism, associated to direct injury or devascularization of the parathyroids in the intraoperative period (3). Thus, most studies have used post-surgery serum levels of parathyroid hormone (PTH) and/or Calcium (Ca) as predictors of hypocalcemia or the need for Ca replacement (4–8). PTH has been the preferred marker as it presents higher accuracy and faster drops after surgery, even in the first three hours PO. This drop also shows similar accuracy in relation to absolute values. The use of Ca as a hypocalcemia predictor has fallen into disuse, partly due to a slower drop time than PTH, with its lowest values between the second and third day PO (9). Although the value of using PTH is established, there is still no consensus on the ideal time to collect the hormone, whether during or after surgery and how long after PO (9).

Magnesium (Mg) is a bivalent ion intrinsically involved in Ca homeostasis. It is absorbed in the intestine and actively and passively reabsorbed in the kidney, as maintaining adequate plasma concentrations of the element is necessary for normal PTH secretion (10). The association between hypomagnesemia and the occurrence of PO hypocalcemia in TT have been documented (11,12). In this sense, the possibility of using Mg as a hypocalcemia predictor has some advantages over PTH, such as lower cost and a much narrower band for normal plasma level in that small variations in its concentration could be sufficient to predict the need for Ca replacement (10).

Given the above, the main objective of our study was to assess the ability of serum Mg concentration, measured on the first PO day, to predict the need for Ca replacement in patients undergoing total and completion thyroidectomy.

## SUBJECTS AND METHODS

This prospective cohort study was approved by the local research ethics committee (CAAE: 67523317.6.0000.5411; protocol: 2.046.729) and all patients were given a clear understanding of the study and signed a free and informed consent form (ICF).

Initially, ninety consecutive patients submitted to TT, in a tertiary hospital, between March 2018 and May 2020, were evaluated. Inclusion criteria were: age between 18 and 85 years, submitted to TT (due to malignant or benign thyroid disease). Exclusion criteria were: cases with uncontrolled hyperthyroidism or hypothyroidism, hyperparathyroidism, undergoing dialysis or using medications that alter serum Ca or Mg concentrations (*e.g.* bisphosphonates, diuretics, cholecalciferol or corticosteroids). Ten patients were excluded, six of them due to the use of medications that altered calcemia and four due to the presence of hyperparathyroidism. Thus, eighty cases were selected and effectively studied.

All surgery was performed by the same team and the standard surgical technique consisted of capsular dissection of the thyroid gland with careful identification and preservation of the parathyroid glands together with the recurrent laryngeal nerve.

General data for sample characterization included gender, age (in years), thyroid volume by ultrasonography (in cm^3^), type of surgery (TT with or without cervical neck dissection) and anatomopathological diagnosis (malignant or benign), as previously reported (13).

The main assessed outcome was the need for Ca replacement during the entire hospital stay and/or after 48 hours and/or 7 days after hospital discharge, while the main variable of interest was serum Mg concentration collected in the morning of the first day PO (Mg1PO). All patients were submitted to the measurement of PTH, thyroid-stimulating hormone (TSH), serum Ca, total proteins and protein fractions (PT), 25-hydroxyvitamin D (25OHD), and serum creatinine (Cr) immediately at admission, and PTH in the first (PTH1h) and eighth (PTH8h) hours after gland removal. Ca and PT measurements were taken every 6h during hospitalization and again 48h and 7 days after hospital discharge.

Patients who evolved with all corrected serum Ca levels (CaC) ≥ 8.0 mg/dL and PTH1h ≥ 11 pg/mL were discharged without Ca replacement therapy. All patients who evolved with absolute values of CaC < 8.0 mg/dL or with PTH1h < 11 pg/mL remained in hospital with Ca monitoring and oral or intravenous Ca replacement as necessary until the condition stabilized. Patients with CaC < 8.0 mg/dL 48h or 7 days after discharge were readmitted for condition stabilization.

Although hypocalcemia was defined as the presence of any measurement of CaC < 8.4 mg/dL during hospital stay and 48h or 7 days after discharge, Ca replacement indicated for those patients who presented CaC < 8.0 mg/dL at those times. Intravenous replacement was indicated for calcemia ≤ 7.5 mg/dL or if there were signs or symptoms related to hypocalcemia. Patients were classified into two groups according to whether patients required Ca replacement or not. These groups were compared for PTH1h, PTH8h and Mg1PO levels as well as the other parameters listed above.

All samples were analysed in the clinical analysis laboratory of the study hospital using the following reference values and methods: 8.4 to 10.2 mg/dL for Ca (spectrophotometry; VITROS Chemistry Products, Rochester, USA), 1.6 to 2.3 for Mg (spectrophotometry; VITROS Chemistry Products, Rochester, USA), 11 to 67 pg/mL for PTH (chemiluminescence; Siemens, Llanberis, United Kingdom), 0.4 a 4.0 μUI/mL for TSH (chemiluminescence; Siemens, Llanberis, United Kingdom), and values > 30 ng/mL for 25OHD (chemiluminescence; Aimara (Abbott), Dublin, Ireland). Payne's formula was used for corrected Ca values (CaC) = total Ca + 0.8 (4-albumin) (14).

### Statistical analysis

Descriptive data analysis was performed with frequency and percentages for qualitative variables and means ± standard deviation or medians with 25^th^ and 75^th^ percentiles for quantitative variables. The Student's *t* test was used to compare variables with symmetric distribution and the Mann-Whitney test for variables with asymmetric distribution. Qualitative variables were compared using the chi-squared and Exact Fisher tests. To assess the predictor variables of Ca replacement throughout the hospital stay and/or after 48 hours and/or 7 days after hospital discharge, a univariate logistic regression was initially performed. Then, using the variables that presented p < 0.25, and after performing the multicollinearity test, a multivariate logistic regression model was created to calculate the isolated predictors of this outcome. ROC (Receiver Operating Characteristic) curves were constructed for variables presenting a significant value in multivariate analysis. Best sensitivity (S) and specificity (E) points were calculated from these for predicting the need for Ca replacement. Finally, the positive predictive value (PPV), negative predictive value (NPV), and accuracy (A) were calculated for this point. All analyses were conducted using SPSS version 23. The best S and E point from the ROC curve was calculated using Medicalc version 14.8.1. The significance level adopted was 5%.

## RESULTS

From the 80 patients studied, 62 (77.5%) were female and mean (± SD) age was 52 (±13) years. Median thyroid volume was 18.6 cm^3^ (minimum and maximum 3.9 and 240 cm^3^ respectively), anatomopathological exam diagnosed malignancy in 45 (56.3%) patients. Twelve (15%) patients were submitted to neck dissection. Ten (12.5%) cases had Mg1PO < 1.6 mg/dL. Fifty (62.5%) patients presented hypocalcemia and 32 (40%) required postoperative Ca replacement. The Ca replacement was made in 27 patients (84.3%) before 48 hours, while five (15.7%) began replacement after 48 hours. Of the 32 patients undergoing calcium replacement, 11 (34.4%) received an intravenous replacement (Table 1). None of the patients with hypocalcemia, with or without Ca replacement, had signs or symptoms consistent with this disorder.

**Table 1:** General data of the studied subjects

Data		
Female n (%)		62 (77.5)
Age (years)*		52 ± 13
Thyroid volume (cm^3^)**		18.6 (11.1; 41.7)
Neck dissection n (%)		12 (15)
Cancer diagnosis n (%)		45 (56.3)
PTH PreO (pg/mL)**		36.1 (26.9; 48.2)
CaC PreO (mg/dL)*		9.4 ± 0.4
25OHD PreO (ng/mL)*		27.3 ± 7.3
TSH PreO (μIU/mL)**		1.8 (1; 2.6)
Cr PreO (mg/dL)**		0.7 (0.6; 0.8)
PTH 1h PO (pg/mL)**		7.6 (3; 25.8)
PTH 8h PO (pg/mL)**		9.5 (3; 25.2)
Mg 1d PO (mg/dL)**		1.9 (1.7; 2)
Mg < 1.6 mg/dL n (%)		10 (12.5)
Hypocalcemia n (%)		50 (62.5)
Ca replacement n (%)		32 (40)
	* Intravenous replacement	11 (34.3)
	* Up to 48 h	27 (84.3)
	* After 48 h	5 (15.7)

* Values expressed as mean ± standard deviation.

** Values expressed as median (25th percentile; 75th percentile). Ca: calcium; CaC: corrected serum calcium; Cr: creatinine; d: day; h: hour, Mg: magnesium; n: number; PO: postoperative; PreO: preoperative; PTH: parathyroid hormone; TSH: thyroid-stimulating hormone; 25OHD: vitamin D.

Median serum concentrations of PTH1h, PTH8h and Mg1PO were higher in without replacement group [17 *versus* (*vs*) 3.0 pg/mL; p < 0.001], (18.2 *vs* 3.0 pg/mL; p < 0.001) and (2 *vs* 1.6 mg/dL; p < 0.001). All patients who had Mg1PO < 1.6 mg/dL were in the group that required Ca replacement (p < 0.001). There was no statistical difference between groups for the remaining studied parameters (Table 2).

**Table 2:** Comparison between patients who progressed with or without the need for calcium replacement postoperatively

Variables		No replacement n = 48	Replacement n = 32	P value
Female n (%)		37 (77.1)	25 (78.1)	0.91
Age (year)*		53 ± 12	50 ± 13	0.36
Thyroid volume (cm^3^)**		20.6 (12.4; 44.6)	15 (8.8; 31.6)	0.22
Neck Dissection n (%)		6 (12.5)	6 (18.8)	0.52
Histopathological diagnosis				0.06
	Cancer n (%)	23 (47.9)	22 (68.8)	
	Benign disease n (%)	25 (52.1)	10 (31.1)	
PTH PreO (pg/mL)*		36.3 (27.6; 48.7)	35.2 (27.2; 46)	0.69
CaC PreO (mg/dL)*		9.4 ± 0.4	9.3 ± 0.5	0.42
25OHD PreO (ng/mL)*		27.7 ± 7.2	26.6 ± 7.4	0.51
TSH PreO (μIU/mL)**		2.1 (1; 2.9)	1.7 (1; 2.4)	0.4
Cr PreO (mg/dL)**		0.7 (0.6; 0.8)	0.7 (0.6; 0.8)	0.89
PTH 1h PO (pg/mL)**		17 (7.6; 34.5)	3 (3; 4)	**< 0.001**
PTH 8h PO (pg/mL)**		18.2 (6.59; 32.5)	3 (3; 3)	**< 0.001**
Mg 1d PO (mg/dL)**		2 (1.9; 2.1)	1.6 (1.5; 1.8)	**< 0.001**
Mg < 1.6 mg/dL n (%)		0 (0)	10 (31.3)	**< 0.001**

* Values expressed as mean ± standard deviation.

** Values expressed as median (25th percentile; 75th percentile). Statistical tests: Student's t, chi-square, Fisher's exact and Mann-Whitney; significance level: p < 0.05. Ca: calcium; CaC: corrected serum calcium; Cr: creatinine; d: day; h: hour, Mg: magnesium; n: number; PO: postoperative; PreO: preoperative; PTH: parathyroid hormone; TSH: thyroid-stimulating hormone; 25OHD: vitamin D.

In univariate analysis, PTH1h, PTH8h and Mg1PO were associated with the need for Ca replacement (Table 3). In multivariate analysis considering anatomopathological diagnosis, and PTH1h, PTH8h and Mg1PO levels, only serum Mg [odds ratio (OR) = 0.0004, 95% confidence interval (95% CI): 0.000003-0.04; p = 0.001] was a predictor of the need for Ca replacement in PO (Table 4).

**Table 3:** Prediction of the need for calcium replacement after total thyroidectomy (univariate logistic regression)

Variables	OR	95% CI	p value
Gender – female	1.06	0.36-3.11	0.91
Age (years)	0.98	0.95-1.02	0.35
Thyroid volume (cm^3^)	0.99	0.98-1	0.48
Neck dissection	1.61	0.47-5.54	0.44
Cancer diagnosis	2.39	0.93-6.1	0.06
PTH PreO (pg/mL)	0.99	0.97-1.02	0.83
CaC PreO (mg/dL)	0.65	0.23-1.82	0.42
25OHD PreO (ng/mL)	0.97	0.91-1.04	0.51
TSH PreO (μIU/mL)	0.89	0.64-1.23	0.5
Cr PreO (mg/dL)	1.7	0.08-36.4	0.73
PTH 1h PO (pg/mL)	0.85	0.78-0.94	**0.001**
PTH 8h PO (pg/mL)	0.85	0.78-0.93	**0.001**
Mg 1d PO (mg/dL)	0.0001	0.000003-0.006	**<0.001**

Statistical tests: univariate logistic regression. Significance level: p < 0,05. CaC: corrected serum calcium; 95% CI: 95% confidence interval; Cr: creatinine; d: day; h: hour, Mg: magnesium; n: number; OR: odds ratio; PO: postoperative; PreO: preoperative; PTH: parathyroid hormone; TSH: thyroid-stimulating hormone; 25OHD: vitamin D.

**Table 4:** Prediction of the need for calcium replacement after total thyroidectomy (multivariate logistic regression)

Variables	OR	CI	p value
Cancer Diagnosis	1.38	0.29-6.57	0.68
PTH 1h PO (pg/mL)	1.01	0.85-1.21	0.84
PTH 8h PO (pg/mL)	0.89	0.73-1.07	0.23
Mg 1d PO (mg/dL)	0.0004	0.000003-0.04	**0.001**

OR: odds ratio; CI: confidence interval; significance level: p < 0,05.

d: day; h: hour; Mg: magnesium; PO: postoperative; PTH: parathyroid hormone.

**Figure 1:**
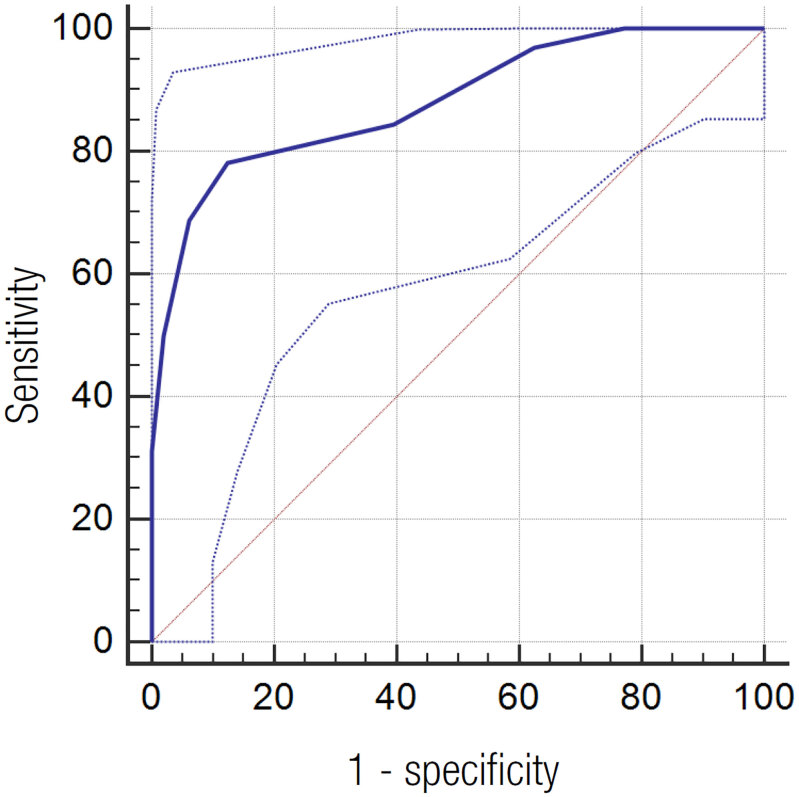
Receiver Operating Characteristic (ROC) curve for the magnesium concentration in the first postoperative day as a predictor of the need for calcium replacement. Area under the ROC curve = 0.88 (95% confidence interval: 0.78 to 0.94, p < 0.0001).

Area under the ROC curve for Mg1PO as a predictor of the need for Ca replacement was 0.88 (95% CI: 0.78 to 0.94; p < 0.0001; Figure 1) and the best S and E cut-off point was 1.8 mg/dL. S, E, PPV, NPV and A for this concentration were 78.1%, 87.5%, 80.6%, 85.7% and 83.7%, respectively.

## DISCUSSION

In this study, it was observed that all patients with Mg1PO < 1.6 mg/dL had hypocalcemia requiring Ca replacement. Additionally, when assessed concomitantly with other factors capable of predicting the need for Ca replacement in PO of TT, Mg1PO was an isolated predictor for replacement. The higher serum concentration of this ion showed as a protective factor against the need for Ca replacement. An absolute Mg value ≤ 1.8 mg/dL on the first PO day presented a sensitivity and specificity of 78.1% and 87.5%, respectively, in predicting the need for Ca replacement during hospitalization and/or on the 48^th^ hour and/or on the 7^th^ day after hospital discharge.

Several studies have looked for predictive factors hypocalcemia or the need for post thyroidectomy Ca replacement, however most are based on falls in Ca and PTH absolute values and percentages between the pre and postoperative period (4–8). However, in recent years, interest has increased in the relationship between the Mg ion and fluctuations in the Ca ion and PTH after thyroid gland removal. Garrahy and cols. retrospectively evaluated 201 thyroidectomy patients from a prospective database and concluded that hypomagnesemia was significantly associated with hypocalcemia and hypoparathyroidism after thyroidectomy (15). Brophy and cols. retrospectively evaluated 173 TT patients with Mg measurements from the 1^st^ and 2^nd^ days PO; they concluded that there was a drop in levels of this ion in these two moments and that its concentration on the 2^nd^ day PO was very strongly related to hypocalcemia (16). Other retrospective studies with larger numbers of patients also reported that hypomagnesemia was significantly associated with postoperative hypocalcemia (11,12).

Some studies have attempted to elucidate the complex inter-relationship between the calcemia and magnesemia balance and serum PTH concentration (10,17–19). In hypercalcemia, a conformational change in CaSR (Calcium sensing receptor) indirectly leads to the recruitment of Gq1 and Gqa subunits of the G protein, changing signalling inside the cell by reducing cAMP levels and activating MAP kinases and phospholipases. These changes rapidly inhibit PTH liberation. However, CaSR is not activated in hypocalcemia, so PTH release is stimulated. PTH promotes a direct increase in calcemia at bone level and an indirect increase at kidney level promoting the conversion of 25OHD into 1,25-dihydroxyvitamin D at intestine level. Mg balance is maintained by renal reabsorption and intestinal absorption of the ion with about 90% occurring passively via the paracellular pathway. However, fine adjustment of serum Mg concentration occurs via renal reabsorption in the distal convoluted tubule through TRPM (transient receptor potential melastatin) subtypes 6 and 7 (19). We know today that small reductions in serum Mg levels stimulates PTH secretion, similar to a drop in calcemia. However, larger drops lead to a paradoxical response inhibiting PTH secretion, leading to hypocalcemia refractory to Ca replacement, but responsive to Mg (18). Despite recent advances in an attempt to elucidate the interrelation between these elements, until now no consensus has been reached from a molecular perspective, as to what the relationship is between falls in Mg and Ca, secondary to a fall in PTH from parathyroid gland injury during thyroidectomy.

In the present study, serum PTH concentrations at 1 and 8 h were higher in the group without Ca replacement and, in the univariate analysis, both were predictors of outcome. However, this finding was not maintained in the multivariate analysis, with only Mg remaining as an isolated predictor. PTH evaluated in the postoperative period has been frequently associated with the prediction of hypocalcemia after TT (20). However, most studies that evaluate this relationship are highly heterogeneous and the ideal moment for this evaluation has not yet been established (21).

One limitation of this study was the relatively low sample number of 80 patients. We believe that an increased number of evaluated cases could have led to the inclusion of factors such as postoperative serum PTH level as a predictor for replacement. Also, failure to measure preoperative serum Mg could be considered another limitation. If this had been done, it could have clarified whether the postoperative drop in this ion was related to the need for Ca replacement. However, this study has merit by demonstrating that the level of serum Mg collected on the first day after surgery presented good sensitivity and specificity in predicting which patients required Ca replacement. Thus, the Mg measurement on the first PO day works as a complementary screening tool, in addition to the already known PTH dosage, to help in the detection of patients that can be safely discharged from the hospital, as they did not require Ca replacement from those who should remain in hospital with monitoring and Ca replacement. Routine use of a single Mg measurement on the first day PO as a marker for Ca replacement after TT has several advantages. The first would be the costs: in our institution, for example, measuring serum Mg is five times less expensive than measuring serum PTH. Also, the many routinely performed Ca measurements would be dispensable thus saving even more as well as saving patients from several serial venous punctures. Another advantage would be the possible detection of hypomagnesemia, a cause of hypocalcemia that may be refractory to Ca and vitamin D replacement, but responsive to Mg (18). Despite the above advantages, the findings of our study still require further study with larger sample numbers and be submitted to validation processes.

Thus, we concluded that serum Mg concentrations collected on the first day after TT, are predictors of the need for Ca replacement, making it an excellent additional tool to screen patients for much earlier hospital discharge after these operations.

## References

[1] Bai B, Chen Z, Chen W (2018). Risk factors and outcomes of incidental parathyroidectomy in thyroidectomy: a systematic review and meta-analysis. PLoS One.

[2] Noureldine SI, Genther DJ, Lopez M, Agrawal N, Tufano RP (2014). Early predictors of hypocalcemia after total thyroidectomy: an analysis of 304 patients using a short-stay monitoring protocol. JAMA Otolaryngol Head Neck Surg.

[3] Edafe O, Antakia R, Laskar N, Uttley L, Balasubramanian SP (2014). Systematic review and meta-analysis of predictors of post-thyroidectomy hypocalcaemia. Br J Surg.

[4] Filho EBY, Machry RV, Mesquita R, Scheffel RS, Maia AL (2018). The timing of parathyroid hormone measurement defines the cut-off values to accurately predict postoperative hypocalcemia: a prospective study. Endocrine.

[5] Puzziello A, Gervasi R, Orlando G, Innaro N, Vitale M, Sacco R (2015). Hypocalcaemia after total thyroidectomy: could intact parathyroid hormone be a predictive factor for transient postoperative hypocalcemia?. Surgery.

[6] Rutledge J, Siegel E, Belcher R, Bodenner D, Stack BC (2014). Barriers to same-day discharge of patients undergoing total and completion thyroidectomy. Otolaryngol Head Neck Surg.

[7] Selberherr A, Scheuba C, Riss P, Niederle B (2015). Postoperative hypoparathyroidism after thyroidectomy: efficient and cost-effective diagnosis and treatment. Surgery.

[8] Vanderlei FA, Vieira JG, Hojaij FC, Cervantes O, Kunii IS, Ohe MN (2012). Parathyroid hormone: an early predictor of symptomatic hypocalcemia after total thyroidectomy. Arq Bras Endocrinol Metabol.

[9] Mazotas IG, Wang TS (2017). The role and timing of parathyroid hormone determination after total thyroidectomy. Gland Surg.

[10] Allgrove J, Shaw NJ (2015). Calcium and Bone Disorders in Children and Adolescents.

[11] Nellis JC, Tufano RP, Gourin CG (2016). Association between Magnesium Disorders and Hypocalcemia following Thyroidectomy. Otolaryngol Head Neck Surg.

[12] Liu RH, Razavi CR, Chang HY, Tufano RP, Eisele DW, Gourin CG (2020). Association of Hypocalcemia and Magnesium Disorders With Thyroidectomy in Commercially Insured Patients. JAMA Otolaryngol Head Neck Surg.

[13] Lloyd RV, Osamura RY, Klöppel G, Rosai J (2017). WHO Classification of Tumours of Endocrine Organs.

[14] Payne RB, Carver ME, Morgan DB (1979). Interpretation of serum total calcium: effects of adjustment for albumin concentration on frequency of abnormal values and on detection of change in the individual. J Clin Pathol.

[15] Garrahy A, Murphy MS, Sheahan P (2016). Impact of postoperative magnesium levels on early hypocalcemia and permanent hypoparathyroidism after thyroidectomy. Head Neck.

[16] Brophy C, Woods R, Murphy MS, Sheahan P (2019). Perioperative magnesium levels in total thyroidectomy and relationship to hypocalcemia. Head Neck.

[17] Quitterer U, Hoffmann M, Freichel M, Lohse MJ (2001). Paradoxical block of parathormone secretion is mediated by increased activity of G alpha subunits. J Biol Chem.

[18] Vetter T, Lohse MJ (2002). Magnesium and the parathyroid. Curr Opin Nephrol Hypertens.

[19] Hoorn EJ, Zietse R (2013). Disorders of calcium and magnesium balance: a physiology-based approach. Pediatr Nephrol.

[20] Le TN, Kerr PD, Sutherland DE, Lambert P (2014). Validation of 1-hour post-thyroidectomy parathyroid hormone level in predicting hypocalcemia. J Otolaryngol Head Neck Surg.

[21] Mathur A, Nagarajan N, Kahan S, Schneider EB, Zeiger MA (2018). Association of Parathyroid Hormone Level With Postthyroidectomy Hypocalcemia: A Systematic Review. JAMA Surg.

